# Kinetic Targeting of pegylated liposomal Doxorubicin: a new Approach to Reduce Toxicity during Chemotherapy (CARL-trial)

**DOI:** 10.1186/1471-2407-11-337

**Published:** 2011-08-04

**Authors:** Jürgen Eckes, Oliver Schmah, Jan W Siebers, Ursula Groh, Stefan Zschiedrich, Beate Rautenberg, Annette Hasenburg, Martin Jansen, Martin J Hug, Karl Winkler, Gerhard Pütz

**Affiliations:** 1 Medical Practice, Altdorfstr. 10, Emmendingen, Germany; 2 Dept. Hematology & Oncology; University Medical Center Freiburg, Hugstetter Str. 55, Freiburg, Germany; 3 Dept. Gynecology, St. Josephs Hospital Offenburg, Offenburg, Germany; 4 Dept. Nephrology; University Medical Center Freiburg, Hugstetter Str. 55, Freiburg, Germany; 5 Dept. of Gynecology and Obstetrics, University Medical Center Freiburg, Hugstetter Str. 55, Freiburg, Germany; 6 Pharmacy; University Medical Center Freiburg, Hugstetter Str. 55, Freiburg, Germany; 7 Dept. Clinical Chemistry; University Medical Center Freiburg, Hugstetter Str. 55, Freiburg, Germany

## Abstract

**Background:**

The therapeutic success of chemotherapeutic agents is often limited by severe adverse effects. To reduce toxicity of these drugs, nanoscale particle-based drug delivery systems (DDS) are used. DDS accumulate to some extent in tumor tissues, but only a very small portion of a given dose reaches this target. Accumulation of DDS in tumor tissues is supposed to be much faster than in certain other tissues in which side effects occur ("Kinetic Targeting"). Once saturation in tumor tissue is achieved, most of the administered DDS still circulate in the plasma. The extracorporeal elimination of these circulating nanoparticles would probably reduce toxicity.

**Methods:**

For the CARL-trial (Controlled Application and Removal of Liposomal chemotherapeutics), pegylated liposomal doxorubicin (PLD) was used as chemotherapeutic agent and double filtration plasmapheresis (DFPP) was performed for extracorporeal elimination of liposomes. PLD was given as 40 mg/m^2 ^every 3 weeks in combination with vinorelbine 2 × 25 mg/m^2 ^(neoadjuvant treatment of breast cancer, 12 patients), or as 40 mg/m^2 ^every 4 weeks (recurrent ovarian cancer, 3 patients). Primary endpoints were the efficiency and safety profile of DFPP, and secondary endpoints were side effects and tumor response.

**Results:**

DFPP eliminated ~62% of circulating PLD, corresponding to ~45% of the total dose (n = 57 cycles). AUC of doxorubicin was reduced by 50%. No leakage of doxorubicin was detected during elimination, and no relevant DFPP-related side effects occurred. Reduction in tumor size > 30% occurred in 10/12 (neoadjuvant) and in 1/3 patients (recurrent). Only five grade 2 events and one grade 3 event (mucositis, neutropenia or leucopenia) and a single palmar-plantar erythrodysesthesia grade 2 were reported.

**Conclusion:**

Extracorporeal elimination of PLD by DFPP is safe and efficient. CARL can diminish the main dose-limiting side effects of PLD, and probably many different DDS alike.

**Trial registration:**

DRKS00000163

## Background

The dosing of chemotherapeutic drugs is a delicate balancing act between killing tumor cells and severe toxicity in other tissues. To reduce the acute adverse effects of toxic drugs, nanoscale particle-based drug delivery systems (DDS) are a very promising approach [1]. Liposomal DDS have been successfully applied in cancer chemotherapy for over a decade. Their *in vivo *behavior and pharmacokinetics are well known [2,3]. Liposomes, like other DDS, accumulate in tumor tissues via the *enhanced permeation and retention effect *[4]. Peak concentrations of liposomal encapsulated chemotherapeutic agents in tumors strongly depend on their initial plasma concentration [5], and the average peak plasma concentration of PLD has been found to correlate closely with the anti-tumor response [6]. In animal models, the elimination of circulating pegylated liposomal doxorubicin (PLD) in plasma is much faster than elimination of (liposomal) doxorubicin from tumor tissue [7]. In humans, a strong signal of liposomal radiotracer was found in tumor tissue after 7 days, while plasma half-life (plasma-t_1/2_) for the liposomal carrier system was estimated as~76 h [8]. Thus minimal or no back diffusion of PLD from tumor tissue to the plasma compartment can be assumed. The tumor compartment is usually much smaller than the plasma or whole body compartment, and despite accumulation, only a tiny portion of a total dose is detectable within the tumor tissue (~0.5 - 3.5% in human tumors [8]). Considering these facts, it is likely that the vast amount of administered DDS is only needed to build up a diffusion gradient between plasma and tumor tissue [9]. Once concentration maximum of liposomes in tumor tissue is achieved, circulating liposomes may contribute to the observed adverse effects, but they may have little further therapeutic value.

PLD has been used worldwide for many years. While the cardiotoxicity of doxorubicin is considerably reduced, severe skin toxicity occurs due to the unique pharmacokinetics of long circulating liposomes [10]. With an approved monotherapy dosage of 50 mg/m^2 ^every 4 weeks (50q4wks), approx. 50% of the patients showed palmar-plantar erythrodysesthesia (PPE, also called hand-foot-syndrome) grade 1, and 20% suffered grade 3 and higher [11]. PPE correlated significantly with plasma-t_1/2 _[12], indicating a slow process. In mouse models, the maximum concentration of doxorubicin (Cmax) in tumor tissues was reached within~24 h, while the maximum concentration in skin and paws was attained after~72 h [7]. Thus there is a distinct time gap in PLD accumulation between tumor tissue and side-effect tissues. Due to the long plasma-t_1/2_, about 70% of administered PLD were still circulating in the plasma 24h after infusion. The concept of Kinetic Targeting takes advantage of distinct accumulation kinetics and long plasma-t_1/2 _of PLD by eliminating a major fraction of circulating DDS once accumulation in tumor tissue has reached its maximum [9].

Size exclusion filtration is the most suitable elimination principle for eliminating various DDS [13], and the respective clinically-used elimination technique known as double-filtration plasmapheresis (DFPP) is well suited for eliminating PLD [14].^. ^In DFPP, the first filtration step separates blood cells from plasma, while a second filtration eliminates particles and high molecular weight components. The system operates continuously. Elimination efficacy depends on the treated plasma volume. On average it takes about 2-3 h to treat one plasma volume and to eliminate~60-70% of circulating particles [15].

This paper describes the first pilot study investigating the concept of Kinetic Targeting. The safety and efficiency of extracorporeal elimination of PLD were addressed in the CARL-trial (Controlled Application and Removal of Liposomal chemotherapeutics) as primary endpoints, and side effects and tumor outcomes were evaluated as secondary endpoints as well.

## Methods

### Patients and Study design

This study was conducted on patients with recently diagnosed primary breast cancer or in patients with recurrent gynecological cancer aged between 18 and 70 years. Their Eastern Cooperative Oncology Group (ECOG) performance status had to be ≤2 and peripheral veins had to permit access of apheresis catheters. Patients were excluded for previous chemotherapy with PLD, extracorporeal treatments or chemotherapeutic drugs other than those proposed. Further exclusion criteria were pregnancy, severe liver, kidney or hematopoetic dysfunction; acute or chronic skin diseases, cachexia/BMI < 20, or acute infection.

This study was conducted as investigator-initiated trial, sponsor was the University Freiburg Medical Center, Dept. of Clinical Chemistry. The study was approved by the Ethics Committee of the University Freiburg (EK-Freiburg 171/07). Written informed consent was provided by all patients prior to participation. The study was registered as DRKS00000163 in the German Registry of Clinical Studies. Participating centers were the University Freiburg Medical Center and St. Josefs Hospital Offenburg. Due to the trial's design, patients were not randomized.

In treatment schedule 1 (TS1), patients with primary breast cancer were treated prior to surgery (neoadjuvant) with 4 consecutive cycles every 3 weeks. Each cycle consisted of vinorelbine (Navelbine^®^) 25 mg/m^2 ^on days 1 and 8, and PLD (Caelyx^®^) 40 mg/m^2 ^on day 15. Plasmapheresis was performed on day 17, 42 - 48 h after infusion of PLD. After two cycles, tumor size was measured by magnetic resonance tomography (MRT), and the therapy was terminated in case of no tumor reduction. Patients who were HER2/neu-receptor positive additionally received trastuzumab (Herceptin^®^) 2 mg/kg/week. Since a small amount of IgG is eliminated during DFPP, IgG in plasma was measured pre-and post-plasmapheresis and the next dosing of trastuzumab was adjusted to the amount of eliminated IgG.

In treatment schedule 2 (TS2), patients with recurrent ovarian or endometrial cancer received PLD (Caelyx^®^) 40q4 wks. Plasmapheresis was performed 44 - 48 h after PLD infusion. Computer tomography (CT) scans were performed every 3 cycles. Response was classified by Response Evaluation Criteria In Solid Tumors (RECIST) [16] and by monitoring tumor marker CA-125.

Primary endpoints were efficiency and safety of DFPP. Efficiency was determined by the eliminated amount of doxorubicin. Overall efficiency was correlated to the initial plasma concentration after infusion of PLD, while DFPP efficiency was correlated to the concentration of circulating PLD at plasmapheresis onset. Doxorubicin in plasma was measured according to the method of Charrois & Allen [7]. Briefly, 500 μl of plasma were diluted with 1 ml of water, 500 μl of 10% (v/v) Triton X-100 and 5 ml of acidified isopropanol (0.75 N HCl) were added. After mixing, the tubes were kept at -22°C for approx. 12 h, then mixed thoroughly and centrifuged at 12.5000 × g for 30 min. Doxorubicin fluorescence was measured using a luminescence-spectrometer LS 50 (PerkinElmer LAS GmbH, Rodgau, Germany). Excitation wave length was 475 nm, emission was detected at 555 nm, slit width was 10 nm.

Criteria for the safety of DFPP were leakage of doxorubicin during filtration and occurrence of plasmapheresis-related side effects. Secondary endpoints were side effects, tumor response and quality of life. Side effects were classified according to international guidelines (CTCAEv3). To assess quality of life (QoL) the EORTC-QLQ-C30 questionnaire was used [17]. The questionnaire was completed prior to chemotherapy and after the last cycle. Scoring was done according to the EORTC guidelines. Data were assessed and scored using the Stata^® ^statistical package Version 11.0 (Statacorp, Texas, USA). To assess the impact of plasmapheresis, a custom-made questionnaire was used (see Table 1).

Total cholesterol was measured enzymatically by CHOD-PAP (DiaSys Diagnostic Systems GmbH, Holzheim, Germany), LDL and HDL-cholesterol were estimated by lipoprotein electrophoresis on SAS Gel Systems (Helena Biosciences, Gateshead, United Kingdom). Plasma protein levels were measured by colorimetric essays (total protein and albumin) or immunological essays (transferring, ferritin, IgG) on a Modular P (Roche Diagnostics GmbH, Mannheim, Germany). CA-125 was measured by immunological essay on a Cobas E 601 platform (Roche).

### Plasmapheresis

As medical device a HF-440 system (Infomed s.a., Genf, Switzerland) or an Octo Nova system (Diamed Medizintechnik GmbH, Cologne, Germany) were used. The filter LF 050 (Infomed) or Plasmaflow OP-05W(L) (Asahi Medical Co. Ltd., Tokyo, Japan) were used as plasmaseparator, and Evaflux 5A or Cascadeflow EC-50W (both manufactured by Kawasumi Laboratories Inc. Tokyo, Japan) were used as particle filter. They showed no differences in performance and are assumed to be identical [14]. The plasma volume to be treated was projected to 3 l. In order to estimate efficiency and safety of the different filtration steps, doxorubicin concentrations in plasma, in the plasma circuit post first filtration step and post second filtration step were measured. Samples were taken prior to plasmapheresis and from every 0.5 l of treated plasma volume. The plasmapheresis system returned the plasma and blood remaining in the extracorporeal unit to the patient once treatment was finished, and the final blood concentration of doxorubicin was measured thereafter. Patients received 2 × 4000 IU heparin as bolus prior to plasmapheresis and 3400 IU heparin/h during plasmapheresis. Heparin infusion was stopped~30 min prior to terminating plasmapheresis.

### Mathematics and Statistics

#### Efficiency of plasmapheresis

Blood was drawn once the infusion of Caelyx was terminated and prior to the plasmapheresis treatment, and plasma-t_1/2 _was calculated according to a monophasic elimination characteristic of first order [2]. To measure the overall efficiency of plasmapheresis, doxorubicin concentration in plasma was estimated immediately prior to the onset of plasmapheresis (c_aphstart_) and after terminating plasmapheresis (c_aphend_). To calculate the eliminated fraction of the total doxorubicin dose, concentration of doxorubicin in plasma immediately after infusion of PLD was estimated (c_0_). Eliminated fraction of total dose (in percent) was calculated by equ. 1, eliminated fraction of circulating dose was calculated by equ. 2.(1)(2)

### AUC of PLD

**Figure 1:**
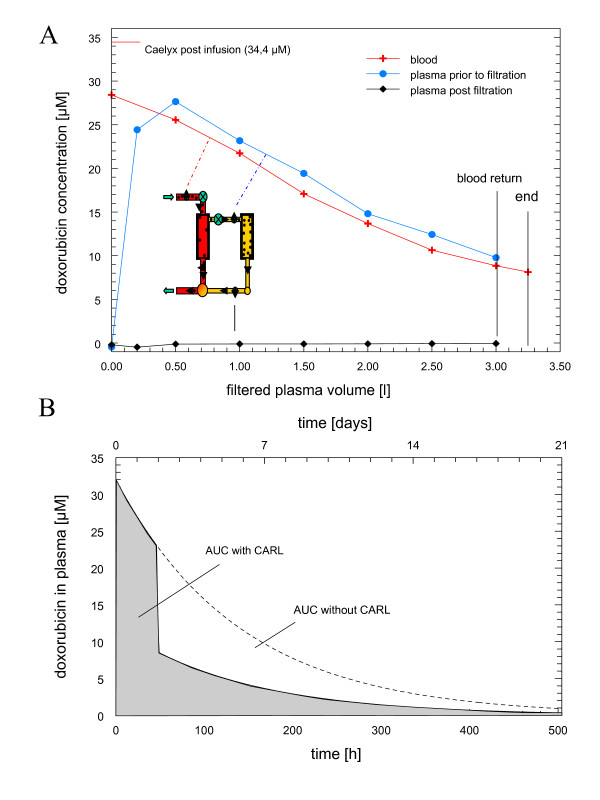
**Apheresis of liposomal doxorubicin**. Chart A shows the concentration of doxorubicin during plasmapheresis in blood (red line), in the separated plasma prior to particle filtration (blue line) and post-particle filtration (black line). The insert shows a schema of the double filtration plasmapheresis used. Blood circuit is red and plasma circuit is yellow. First filtration separates plasma from blood cells, second filtration step eliminates particles. The plasmapheresis system returns the plasma and blood remaining in the extracorporeal unit to the patient once treatment is finished, and final blood concentration of doxorubicin was measured thereafter. Average blood flow rates were between 50 and 65 ml/min. A typical plasmapheresis is shown (n = 57). Chart B shows the estimated area under the plasma concentration curve (AUC) for liposomal doxorubicin without plasmapheresis (below dashed line) and with plasmapheresis (gray).

The area under the plasma concentration curve (AUC) of doxorubicin was calculated for each cycle (figure 1B). Plasma-t_1/2 _of PLD until plasmapheresis was calculated by c_0 _and c_aphstart_. The (hypothetical) AUC without plasmapheresis was calculated according to c_0 _and estimated plasma-t_1/2 _for the full length of the cycle. AUC with plasmapheresis was calculated according to c_0 _and plasma-t_1/2 _until the start of plasmapheresis and according to c_aphend _from the end of plasmapheresis until the next cycle (for details see additional file 1).

## Results

### Patients

Between February 2008 and November 2010, and 15 patients were enrolled. Patient age was between 39 and 67 years (median 53). Of 15 patients in this study, 12 patients were treated for breast cancer. Their characteristics are shown in table 1, 6 patients were HER-2-neu positive and received trastuzumab additionally. Due to no response or persistent inflammatory disease, one patient received only 2 and one patient only 3 cycles of chemotherapy. All other 10 patients underwent 4 cycles of TS1. In TS2, 2 patients showed progressive disease after the 3rd cycle and were switched to an alternative regimen; 1 patient with recurrent ovarian cancer regressed and received 6 cycles of TS2. All cycles were delivered as planned.

**Table 1:** Patients treated for primary breast cancer

n	12
Age (median and range)	53 (39-67)
Staging	
IIA	2
IIB	4
IIIA	2
IIIB	4
Receptor	
ER/PR +/+	4
ER/PR +/-	6
ER/PR-/-	2
HER2/neu +	6

Staging was done according to the American Joint Committee on Cancer TNM system. ER: Estrogen receptor; PR: progesterone receptor

### Plasmapheresis

A total of 57 plasmapheresis treatments were performed, with 56 treatments achieving the projected plasma volume. One plasmapheresis treatment had to be terminated prematurely due to clotting in the vein-access catheters. All other treatments went smoothly. Average blood flow rates of the therapies were between 50 and 65 ml/min. A typical plasmapheresis treatment is shown in figure 1A. Blood concentration dropped nonlinearly due to continuous reperfusion. The concentration of doxorubicin in plasma was equal to the respective blood concentration, indicating that liposomes pass the plasma filter membrane without restriction. In contrast, the doxorubicin concentration post particle filter was close to baseline, indicating that all liposomes were retained in the second filter system. Most importantly, no doxorubicin was leaking out of the liposomes during the filtration process.

The average elimination characteristics of doxorubicin are given in table 2. Plasmapheresis onset was 42-48 h (average 46 h) after terminating the PLD infusion. At that time point,~72% (± 6%) of the initial doxorubicin dosage were found in circulation. About 62% (± 9%) of the circulating PLD were eliminated by extracorporeal purification of 3l of plasma, yielding an average elimination of 45% (± 7%) of the total doxorubicin dose. Thus plasmapheresis of PLD can be considered very efficient under the conditions applied.

**Table 2:** Elimination of doxorubicin by double filtration plasmapheresis

C_max _of doxorubicin post infusion	34 ± 5	[μM]
Doxorubicin at plasmapheresis onset	72 ± 6%	[% of C_max_]
Doxorubicin at plasmapheresis termination	28 ± 9%	[% of C_max_]
Elimination of total doxorubicin	45 ±7%	[% of total doxorubicin dose]
Elimination of doxorubicin in plasma	62 ± 9%	[% of plasma doxorubicin at plasmapheresis initiation]
Reduction in AUC	50 ± 3%	

A total of 57 apheresis treatments were conducted. Treated plasma volume was 3 l, plasmapheresis was initiated~46 h after PLD infusion was terminated. Concentration of doxorubicin in plasma was measured immediately after infusion (c_max_), at apheresis onset and when apheresis was terminated. The eliminated amount of total doxorubicin dosage (line 4) and of circulating liposomal doxorubicin at apheresis onset (line 5) is given. The AUC was calculated as described in methods, and the reduction in AUC of doxorubicin by apheresis is stated. Data are mean ± SD.

Elimination of lipoproteins and main plasma proteins were measured as well (see table 3). Elimination of plasma lipids and proteins was in the normal range for DFPP by the materials used [18]. Within the 3-4 weeks until the next plasmapheresis, plasma levels of lipids and proteins had reached their initial value, and no general decline occurred during the 4 to 6 cycles of chemotherapy. In general, plasmapheresis was tolerated very well, with only one plasmapheresis-related side effect, namely a mild drop in blood pressure, during 57 treatments.

**Table 3:** Elimination of plasma lipids and proteins

Plasma component	plasma level (prior to plasmapheresis)	elimination [%]
Lipids		
Total cholesterol	193 ± 33 [mg/dl]	46 ± 8
LDL-cholesterol	104 ± 27 [mg/dl]	59 ± 10
HDL-cholesterol	57 ± 22 [mg/dl]	10 ± 8
Proteins		
Total protein	6,7 ± 0,3 [g/dl]	16 ± 3
Albumin	4,2 ± 0,2 [g/dl]	13 ± 4
Transferrin	233 ± 30 [ng/dl]	12 ± 4
Ferritin	152 ± 115 [ng/dl]	22 ± 7
IgG	848 ± 144 [mg/dl]	18 ± 5

Plasma levels were measured by standard clinical chemistry methods immediately prior and past plasmapheresis. Fasting was not required prior to plasmapheresis, so total cholesterol, LDL-cholesterol and HDL-cholesterol could not be accurately estimated in all cycles (n = 32-57). All values are mean ± SD.

### Doxorubicin pharmacokinetics

C_max _of doxorubicin post-infusion was 34 ± 5 μM. The overall plasma-t_1/2 _of PLD was 93 (± 12) h, slightly above earlier data [2]. In order to restrict total blood loss for patient safety, pharmacokinetics were estimated by the most essential data points only, and the discrepancy in plasma-t_1/2 _to previous published data is probably due to few data points available. Mean plasma-t_1/2 _were 90 (± 18), 93 (± 17), 105 (± 25) and 119 (± 37) h for cycle 1 to 4. Of 11 patients receiving > 3 cycles, 6 showed a consecutive progression of plasma-t_1/2_, while 5 patients showed no tendency for prolonged plasma-t_1/2_. The reduction in total doxorubicin dosage was 45%. Impact of plasmapheresis on AUC was roughly estimated for each cycle by the measured plasma-t_1/2 _and DFPP efficiency (details see additional file 1). AUC with plasmapheresis was 50% (±3%) of the calculated AUC without plasmapheresis (figure 1B)

### Toxicity

**Figure 2:**
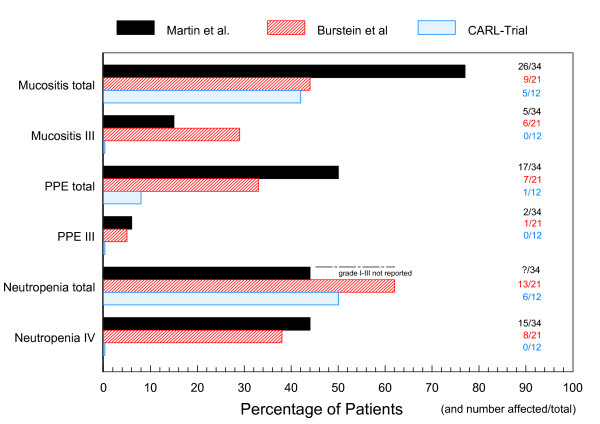
**Major toxicities observed during the CARL trial and related published trials**. The major levels of toxicity for different treatment schedules using a combination of PLD and vinorelbine are given as percentage of patients and as number of patients/total patients in trial. Grading according to CTCAEv3-criteria is given in Roman numerals. Black bars: study of Martin et al. (n = 34), vinorelbine 30 mg/m^2 ^and PLD 35 mg/m^2 ^every 4 weeks [20]; red bars: study of Burstein et al. (n = 21), vinorelbine 2 × 25-30 mg/m^2 ^and PLD 40-50 mg/m^2 ^every 4 weeks [19]; blue bars: CARL-Trial (n = 12), vinorelbine 2 × 25 mg/m^2 ^and PLD 40 mg/m^2 ^+ plasmapheresis every 3 weeks. Unfortunately, the frequency of neutropenia < grade IV is not reported in the study of Martin et al.. PPE = palmar-plantar erythrodysesthesia, PLD: pegylated liposomal doxorubicin.

The most remarkable result of the CARL pilot trial was the occurrence of a single grade 2 PPE event during 57 cycles of PLD (figure 2). The same patient reported a grade 1 exanthema, the only reported exanthema during the trial. This result is even more remarkable as the average dosage of PLD was slightly higher in TS1 (13,3 mg/m^2^/week) than in the standard regimen 50q4 wks (12,5 mg/m^2^/week). Side effects were graded according to CTCAEv3 guidelines, parentheses show the numbers of cycles affected and the respective percentage according to the total number of cycles in TS1 or TS2. In TS1 mucositis/stomatitis grade 1 were reported by 4 patients (8 cycles, 18%), and grade 2 by 1 patient in 1 cycle (2%). Six patients reported fatigue (12 cycles, 27%) in TS1 and 2 patients (4 cycles, 33%) in TS2. Alopecia was reported after 2 cycles (4%) by 1 patient in TS1. One patient in TS2 presented grade 1 obstipation during 3 cycles, another patient had diarrhea during 1 cycle and nausea grade 1 after the first PLD infusion. In TS1, six patients had grade 1 leucopenia (17 cycles, 38%) while two patients had grade 2 leucopenia during 3 cycles (7%). Four patients suffered neutropenia grade 1 (11 cycles, 23%), and one patient grades 2 and 3 (1 cycle each, 2%). In TS2, no hematological toxicology was reported. No significant increase in safety parameters and proBNP-levels were monitored during the study except for a slight increase in liver enzymes (grade 1) during 1 cycle in TS2.

### Response

One reason to include patients with neoadjuvant treatments in this pilot study was the opportunity to assess the size of the primary tumor as a direct readout of clinical response. Of 12 patients included on TS1, 10 patients showed a response. Nine patients had a significant reduction of tumor size > 50%, with two patients > 90%. Average tumor size reduction was~70%. Breast-conserving surgery was successful in 6 patients who completed TS1. For the three patients with a gynecological tumor on TS2, one showed remission and a decline in CA-125 from 99 to 75 U/ml while 2 patients showed progredient lesions and an increase in CA-125.

### Quality of life and acceptance

**Figure 3:**
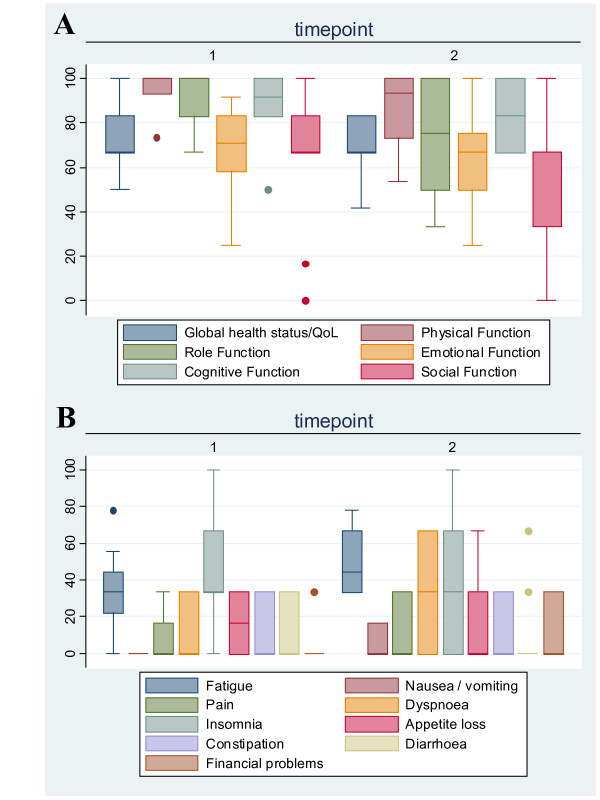
**QLQ-C30 Scores**. To address quality of life during chemotherapy, the QLQ-C30 questionnaire was used [17]. The scores were calculated according to the manual. The questionnaire was filled after the first cycle (timepoint 1) and after the last cycle (timepoint 2), 10 patients were eligible completing 4 (n = 9) or 6 (n = 1) cycles. (**A**) Functional scores (high score corresponds to high functionality) (**B**) Symptom scores (low score correspond to mild symptoms).

To address the impact of chemotherapy on QoL, the questionnaire QLQ-C30 [17] was completed after the first and last cycles. Altogether 10 patients were eligible for data assessment, 9 patients after 4 cycles (TS1) and 1 patient after 6 cycles (TS2). The results are shown in figure 3. Due to the low number of patients, a statistical analysis was not feasible. In general, functional scores remained high during chemotherapy (all medians > 50%), while all symptom scores remained low (all medians < 50%) While the medians of the functional parameters remained more or less unchanged during chemotherapy, few individual scores declined as indicated by a wider range in percentiles. Interestingly, global health status and overall QoL did not change between first and last cycles according to the patients' self-ssessment. In general, the combinations of PLD and plasmapheresis only had a slight impact on patient-reported QoL. To evaluate the impact of extracorporeal treatment during chemotherapy in more detail, we used a customized questionnaire. In general, most of the patients felt well, prior to, during and after plasmapheresis (see table 4).

**Table 4:** Questionnaire Plasmapheresis

How did you feel ...	very good	good	moderately	poor
- prior to plasmapheresis	1	36	11	2
- during plasmapheresis	1	40	7	2
- immediately after plasmapheresis	1	41	6	2
in between plasmapheresis and next cycle (not assessed after last cycle)	0	25	9	2
	no	little	moderate	high
Classify your effort for plasmapheresis treatment today	8	25	10	7

To asses any direct impact of plasmapheresis on Quality of life, a custom-made questionnaire was given to the patients after each cycle. 50 cycles in total were evaluated. No significant change between different cycles became apparent.

## Discussion

The CARL trial is the first clinical trial to investigate the principle of Kinetic Targeting by combining DFPP and PLD. DFPP has been used in clinical practice for years to eliminate natural plasma components. PLD is the most widespread anticancer drug using a nanoscale DDS, and has a very favorable pharmacokinetic profile for Kinetic Targeting. Besides monotherapy, PLD has been successful in various combinations, and the combination of vinorelbine and PLD has proven to be highly efficacious with tolerable side effects [19,20]. PLD was initially approved as monotherapy 50q4 wks, but due to the fear of side effects, a lower dosage of 40q4 wks is commonly used in clinical practice [21]. In this study, a low dose monotherapy (TS2) in recurrent disease and a dose-intensive combination of vinorelbine and PLD as neoadjuvant treatment were evaluated (TS1). Since first treatments on TS1 showed minimal side effects, the ongoing study focused on the dose-intensive treatment, and a total of 12/15 patients were enrolled on TS1. The protocol for TS1 was based on a protocol investigated in the study center in Offenburg, containing 35 mg/m^2 ^PLD on day 15. Despite Kinetic Targeting, plasmapheresis may reduce the amount of accumulated drug in tumor slightly. When a model calculation was performed (see additional file 2), a dosage 40 mg/m^2 ^+ plasmapheresis showed higher Cmax in tumor than a dosage of 35 mg/m^2 ^without plasmapheresis. Beside Cmax, AUC in tumor rose by~2%, while Cmax and AUC in skin fell by~20%. Thus the dosage used in TS1 was raised to 40 mg/m^2 ^PLD.

Primary endpoints of the CARL pilot trial were the efficiency and safety of PLD elimination by DFPP. Just one mild plasmapheresis-related side effect was noted in 57 plasmapheresis treatments. Efficiency of PLD elimination was as high as known for other DFPP procedures. Most importantly, neither liposomal nor free doxorubicin were detected in the plasma beyond the particle filter, indicating that PLD can be filtered without leakage even after prolonged circulation in vivo. Safety and efficiency of PLD elimination by DFPP were considered excellent in this trial.

The most striking result of the CARL pilot trial was the occurrence of a single grade 2 PPE during 57 cycles of PLD. Interestingly, the elimination efficiency for the patient reporting PPE after the 3^rd ^cycle was significantly below average (32-36% of total dose). The pilot study data are even more promising since 12 patients received 13,3 mg/m^2^/week of doxorubicin in a combination therapy, slightly more than the recommended dosage for monotherapy (12,5 mg/m^2^/week). At recommended dosage, approx. 50% of the patients showed PPE grade 1 and 20% grade 3 and higher [11]. In a combination of vinorelbine and PLD 35q4 wks, 49% developed PPE and 62% mucositis [20]. Furthermore, the maximum tolerated doses for PLD in different combination therapies on a 3-doses-a-week schedule were 30 mg/m^2 ^[22], 35 mg/m^2 ^[23] or 45 mg/m^2 ^with PPE/mucositis occurring at 40 mg/m^2 ^[24]. Besides one grade 2 PPE only five grade 2 and one grade 3 events were observed during 45 cycles of the dose-intense TS1 in the CARL-trial. No dose modification or any delay was necessary to manage toxicity. In combination with plasmapheresis, PLD at a higher dosage than originally recommended for monotherapy was very well tolerated in combination with vinorelbine. In the low dosage TS2, we noted only minor side effects and just grade 1 events. While antitumor efficacy is correlated to Cmax [6], PPE correlated significantly to plasma-t_1/2 _[12]. DFPP induces a significant cut in plasma-t_1/2_, and the observed absence of PPE is well in line with these prior observations. In a certain sense, CARL may be considered as a means of cutting plasma-t_1/2 _while keeping Cmax constant.

Most patients accepted plasmapheresis treatment very well, as reflected in the questionnaires (table 4).

In TS1, response was observed in 10/12 patients, and 9/12 patients experienced a tumor size reduction > 50%, in line with other highly efficient anthracycline containing protocols in neoadjuvant treatment of breast cancer [25-27]. Response rates in recurrent platinum-resistant ovarian cancer with PLD 40q4 wks as second line treatment are generally low [28]. With 3 patients in TS2, we observed one partial response. These data indicate that PLD was still active on the low dosage schema despite scheduled extracorporeal elimination of circulating drug. While the reduction of side effects was apparent, we cannot address therapeutic efficacy due to this pilot trial's small cohort. A randomized phase II trial with sufficient statistical power is clearly needed to address this question.

Within the breast cancer group, 6/10 patients were HER2/neu-positive and received trastuzumab. The combination of trastuzumab and PLD has shown encouragingly low cardiac toxicity in these patients, but also an elevated incidence of PPE. Combining trastuzumab with PLD at 50q4wks, the incidence for PPE grades 1-2 was 37% and for PPE grade 3 even 30% [29]. PLD at 40q4wks led to PPE grades 1 and 3 in 12,5% of patients respectively [30]. A combination of trastuzumab with PLD (30 mg/m^2^) and docetaxel (60 mg/m^2^) every 3 weeks led to 75% PPE (38% grade 3) [26]. Combining these data and the lack of any PPE in our 6 patients receiving high dosage of PLD and trastuzumab, patients with HER2/neu-positive metastatic breast cancer may have an additional significant benefit by using a combination of trastuzumab, PLD, and plasmapheresis.

The approved dosage for PLD monotherapy is 50q4wks, but it was assumed that a reduced dosage of 40q4wks would reveal less toxicity while maintaining clinical efficacy [21]. While the benefit in side effects seems obvious, the assumption of maintaining clinical efficacy was based on two retrospective studies and a single phase-II prospective trial with a very low response rate (9%) [24]. All those studies were performed on patients with recurrent ovarian cancer who generally show a low response rate. The rationale behind the dose reduction was an obvious improvement in quality of life, while any impact on clinical benefit remained hidden behind the curtain of statistical power. On the other hand, an early study by Muggia et al. [31] showed a high response rate (27%) for patients with platinum -resistant recurrent ovarian cancer using a high dosage of 50q3wks, but they also found an intolerable incidence of side effects, mainly PPE and stomatitis. Although there is no prospective study comparing 50 to 40 mg/m^2^, the lower dosage has been widely accepted in clinical practice due to the fear of PLD side effects. Kinetic Targeting is a much smarter variant of dose modification, and due to this trial's data, we propose a smarter way of minimizing toxicity and a lesser impairment of clinical efficacy by CARL than by a simple dose reduction. Correlating tumor response to dosage is often difficult. PLD accumulation in tumor tissue is strictly dependent on Cmax [5,7,32]. In patients with Kaposi's sarcoma, average peak plasma concentrations as well as dose intensities have been found to correlate very closely with anti-tumor response [6]. The same was seen in animal models, where a single bolus was more effective than administrating equal amounts of drug in split portions [32,7]. Thus reducing the recommended dosage in mono- or combination therapy is probably paid by disproportionate high reduction in anti-tumor efficacy [33]. Given the diminished incidence of PPE in this trial, maintaining recommended dosage in combination therapies or even a more intensive dosage in monotherapy might become feasible (e.g. 50q3wks) and should be evaluated in future.

Severe side effects often cause the next cycle to be delayed, even though maintaining dosage is crucial for therapeutic success. In the study of Burstein et al. [19], 18/73 cycles were delayed or vinorelbine omitted, and 7/18 patients required a dose reduction in the cohort receiving PLD 40q4wks. In the study reported by Lyass et al, [12] only~25% of patients received the fourth dosage on time when PLD was given as monotherapy 35q3wks. Within the CARL trial, all patients received their recommended dosage on time, even though 45 cycles were given on the dose-intense TS1. Keeping chemotherapy on schedule is another likely benefit of CARL, probably improving the clinical efficacy of the proposed treatments.

The CARL trial is the first clinical trial investigating the principle of Kinetic Targeting, and demonstrating the feasibility of this approach. We used currently available and approved materials and DDS. DFPP is based on size exclusion, and is generally applicable for all kinds of nanoparticle based DDS having the appropriate size between approx. 500 and 25 nm. Fortunately, the nanoparticles used in anticancer treatment are usually in that size range [34]. For Kinetic Targeting a long plasma-t_1/2 _is needed, a basic feature of most nanoparticles investigated so far, and the technique used should be applicable with a broad range of nanoparticle-based DDS. PLD is optimized in terms of plasma-t_1/2_, but not regarding the bioavailability of drug delivered to the tumor. Several improved DDS are being investigated preclinically, and some in clinical trials [34]. All these approaches deal with the DDS reaching the tumor, but CARL deals with the major fraction of DDS not reaching the tumor. Thus we propose that CARL may prove especially valuable with future DDS, revealing improved targeting and release properties.

## Conclusion

DFPP is an efficient and safe technique to eliminate PLD during chemotherapy. The combination of PLD and DFPP allowed successful therapy and a clearly favorable side-effect profile. In contrast to a simple dose reduction, the concept of Kinetic Targeting enables profound drug elimination at a time point when the impact on adverse toxicity is likely to be much greater than the impact on tumor toxicity.

## List of Abbreviations

AUC: area under the plasma concentration curve (AUC); CARL: Controlled Application and Removal of Liposomal chemotherapeutics; Cmax:maximum concentration of doxorubicin; CT:computer tomography; DDS: drug delivery systems; DFPP: double-filtration plasmapheresis; MRT: magnetic resonance tomography; plasma-t_1/2 _: plasma half life; PLD: pegylated liposomal doxorubicin; PPE: palmar-plantar erythrodysesthesia; QoL: quality of life; TS1/2: treatment schedule 1 or 2; XqYwks: × mg/m^2 ^doxorubicin every y weeks

## Conflicts of interest

The authors JE, OS, MJH and GP founded Telltargeting Medical GmbH, a start-up company that holds a patent on the basic principle of scheduled elimination of drug delivery particles.

## Authors' contributions

The authors GP, JE, OS, MJH and KW developed the trial outline, protocols and applied for ethics committee approval. The authors GP, JE and OS organized the trial, while KW was responsible for funding and formalities. The authors JWS and UG were responsible for chemotherapy and patient care in the Offenburg study center, and the authors BR and AH in the Freiburg study center. OS and SZ were responsible for patient information and obtaining written consent, while SZ was responsible for the extracorporeal technique during the treatments. Data analysis was mainly done by GP, JE and KW. The model calculation was done by MJ. The manuscript was written by GP as corresponding author, while the authors AH, MJH and JE made major contributions and improvements. All authors read and approved the final manuscript.

## Pre-publication history

The pre-publication history for this paper can be accessed here:

http://www.biomedcentral.com/1471-2407/11/337/prepub

## Supplementary Material

Additional file 1 **Estimation of AUC**. This file contains a detailed description about the calculation of AUC. Click here for file

Additional file 2 **Model calculation about the impact of plasmapheresis**. This file contains a model calculation based on the data known from animal studies [7]. The model tries to simulate the impact of plasmapheresis on tumor accumulation of PLD. Two scenarios were calculated, one scenario regarding the EPR-effect and one scenario using a simple equilibrium model. Click here for file
