# Supplementary material for: Kinetic Targeting of pegylated liposomal Doxorubicin: a new Approach to Reduce Toxicity during Chemotherapy (CARL-trial)
Source: BMC Cancer. 2011 Aug 4;11:337. doi: 10.1186/1471-2407-11-337 (PMC3175222; doi:10.1186/1471-2407-11-337)
Supplement: Additional file 1 — Estimation of AUC. This file contains a detailed description about the calculation of AUC. [file 1471-2407-11-337-S1.PDF]

Additional file 1

Estimation of AUC

For each cycle, AUC was calculated. Concentration of doxorubicin was estimated in plasma immediately after infusion of PLD (c_0), prior to onset of apheresis ($c_{aphstart}$) and after termination of apheresis (c_{aphend}). Elimination of Caelyx was assumed first order and monophasic[2] (equ. 3).

$$c(t) = c_0 \cdot \exp(-\frac{1}{k}t) \qquad (3)$$

With

$$k = \frac{t_{1/2}}{0.693} \qquad (4)$$

The constant k was estimated by regression analysis using the measured doxorubicin concentrations c_0 and $c_{aphstart}$. Regression was done iterative by slide write curve fitting function (Slide Write 6.0, Advanced graphics software Inc., California, USA).

To calculate the AUC, the respective integrals of equ. 3 were used. To calculate AUC without apheresis (AUC_{normal}), the integral between t=0 and t=504 (3 weeks = 21 days =504 h) was estimated.

$$AUC_{normal} = \int_0^{504} c_0 \cdot -k \cdot \exp(-\frac{1}{k}t) \qquad (5)$$

For calculating of the AUC with apheresis (AUC_A), the AUC of three distinct phases were summarized:

$$AUC_{apher} = AUC_{apher1} + AUC_{apher2} + AUC_{apher3} \qquad (6)$$

AUC_{apher1} corresponds to the AUC until the onset of apheresis, AUC_{apher2} corresponds to the AUC during the time of apheresis, and AUC_{apher3} describes the AUC from termination of apheresis until end of cycle.

With $t_{aphstart}$ being the time in hours until apheresis was initiated, AUC_{apher1} was calculated in the same way as AUC_{normal}:

$$AUC_{apher1} = \int_{0}^{t_{aphstart}} c_0 \cdot -k \cdot \exp(-\frac{1}{k} t) \qquad (7)$$

With $c_{aphstart}$ being the plasma concentration of doxorubicin at apheresis onset, c_{aphend} being the respective concentration when apheresis was terminated, and t_{apher} being the duration of apheresis (~3-4 h), AUC_{Phase2} was calculated:

$$AUC_{apher2} = (c_{aphstart} - c_{aphend}) \cdot t_{apher} \qquad (8)$$

When apheresis was terminated, elimination was assumed to proceed normal, and AUC_{apher3} was calculated from $t=t_{aphend}$ until the end of cycle (t=504 h) using c_{aphend} and k.

$$AUC_{Phase3} = \int_{t_{aphend}}^{504} c_{aphend} \cdot -k \cdot \exp(-\frac{1}{k} t) \qquad (9)$$

The differences in AUC were calculated as:

$$\Delta AUC = \left(1 - \frac{AUC_{Apher}}{AUC_0}\right) \cdot 100 \ [\%] \qquad (10)$$
