# Supplementary material for: Kinetic Targeting of pegylated liposomal Doxorubicin: a new Approach to Reduce Toxicity during Chemotherapy (CARL-trial)
Source: BMC Cancer. 2011 Aug 4;11:337. doi: 10.1186/1471-2407-11-337 (PMC3175222; doi:10.1186/1471-2407-11-337)
Supplement: Additional file 2 — Model calculation about the impact of plasmapheresis. This file contains a model calculation based on the data known from animal studies [7]. The model tries to simulate the impact of plasmapheresis on tumor accumulation of PLD. Two scenarios were calculated, one scenario regarding the EPR-effect and one scenario using a simple equilibrium model. [file 1471-2407-11-337-S2.PDF]

Additional file 2

Model calculation about the impact of plasmapheresis

To make a rough estimate about the impact of plasmapheresis on doxorubicin in tumor tissue, model calculations were performed prior to initiating the study.

The calculations were based upon the data of Charrois & Allen [7] about accumulation of PLD in tumor tissue and in skin and paws of mice. Plasma-$t_{1/2}$ of PLD was estimated to 40 h. For fitting, the program Scilab 5.1.1. and the Downhill Simplex Algorithm were used. First a simple two compartment model was calculated (plasma compartment and tissue compartment, no reflux from tissue compartment). Doxorubicin concentration was calculated according to equ. 11 and 12. In the two compartment model, the concentration maximum of doxorubicin (Cmax) in the tissue compartment is reached within ~72 h. This behavior corresponds very well to the accumulation observed in skin and paws, thus this model was used to calculate doxorubicin concentration in paws (see fig.4).

$$\frac{\partial C_{plasma}}{\partial t} = -C_{plasma} * d_1 \qquad (11)$$

$$\frac{\partial C_{paws}}{\partial t} = -C_{paws} * d_3 + k_{paws} * C_{plasma} \qquad (12)$$

In equitation 11, d_1 relates to elimination in plasma and corresponds to plasma-$t_{1/2}$. The constants d_3 and k_{paws} correspond to elimination and accumulation in skin and paws. They were fitted by regression analysis with start values C_{plasma}=bolus and C_{paws}=0.

EPR-effect model

Tumor accumulation of PLD is much faster than accumulation in other tissues due to the EPR-effect, and the simple two compartment model does not correspond to fast accumulation. To take account of the EPR-effect, the following equitation was used to estimate tumor concentration:

$$\frac{\partial C_{tumor}}{\partial t} = -C_{tumor} * d_2 + k_{tumor} * C_{plasma} * \left(\frac{C\max_{tumor} - C_{tumor}}{C\max_{tumor}} \right) \qquad (13)$$

The constants d_2 and k_{tumor} correspond to elimination and accumulation in tumor tissue. They were fitted by regression analysis with start values C_{plasma}=bolus and C_{tumor} =0. Cmax was given by the data [7]. Not surprisingly, d_2 and d_3 were merely identical and slow, while k_{tumor} was much higher than k_{paws}. The restriction for accumulation depending on already accumulated PLD (equ. 14) was introduced in equ. 13 to account for early accumulation maximum.

$$F = \left(\frac{C\max_{tumor} - C_{tumor}}{C\max_{tumor}} \right) \qquad (14)$$

Fig. 4 shows the accumulation over time for tumor and paws using equ. 13 and 12 (Bolus 1,00, red line). Accumulation maximum was reached after ~34 h.

As generally accepted, $Cmax_{tumor}$ is a linear function of peak plasma concentration [5,7]. The black line in fig. 4 shows the accumulation when the dosage is increased by 14% (e.g from 35 to 40 mg/m^2). In order to simulate plasmapheresis after 24h, the plasma concentration was reduced by 65% at t=24 h. The respective accumulation in case of increased dosage and plasmapheresis is given as dashed blue line in fig. 4. Increased dosage and plasmapheresis lead to an increase in peak tumor concentration of 13% and an overall increase in AUC by 2%.

Accumulation of nanoparticles in tumor tissue is still poorly understood, thus a detailed model is lacking. The model calculation used takes into account that there is no back diffusion from tumor compartment to plasma as proposed by the EPR-effect and considers that “refill” of eliminated liposomes by long circulating liposomes is reduced by plasmapheresis.

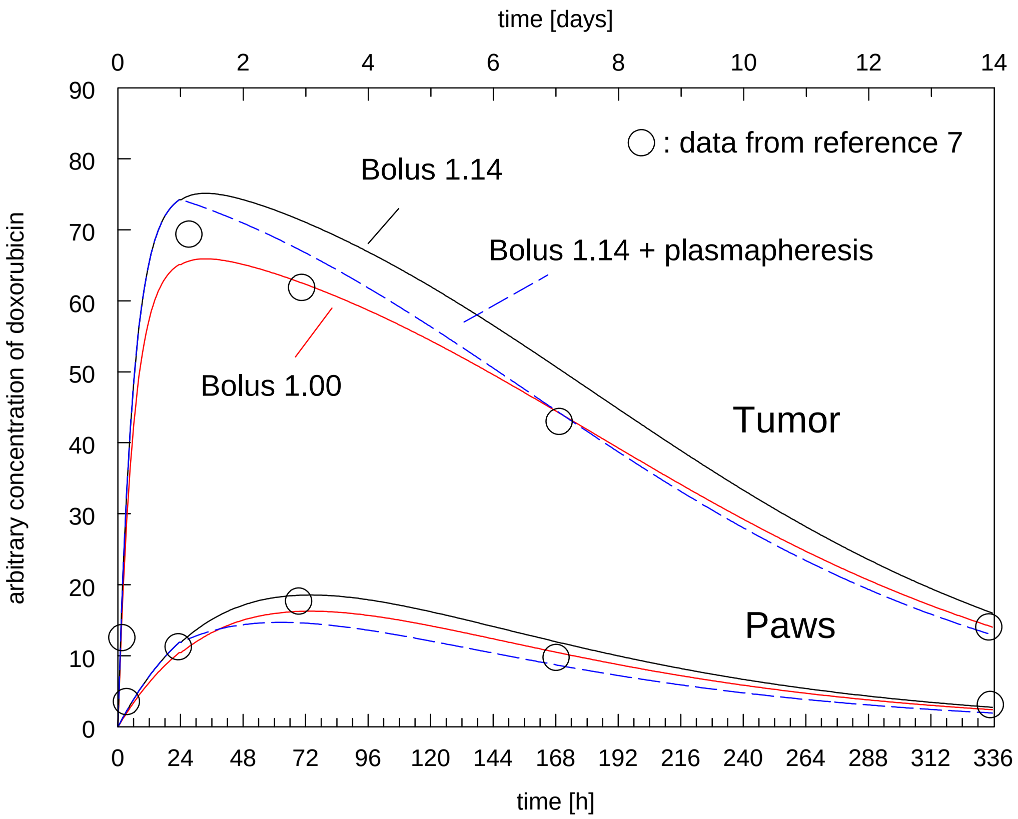

Fig. 4. EPR-effect model. The picture shows the simulated time course of doxorubicin concentration in tumor and in paws according to equ. 11, 12 and 13. The red line shows the fitted model according to the data provided by Charrois & Allen [7], shown as open circles. The simulated bolus (= peak plasma concentration) was set to 1.00 to fit the data. The black line shows a simulation using the fitted constants, but bolus and $Cmax_{tumor}$ were increased by 14% (corresponding to an increase from 35 to 40 mg/m^2). The dashed blue line corresponds to a bolus of 1.14 and plasmapheresis after 24 h decreasing plasma concentration by 65% after 24 h.

Equilibrium model

Despite being in contrast to EPR-effect, a rapid equilibrium between plasma and tumor compartment 1 and slow irreversible accumulation from tumor compartment 1 to tumor compartment 2 was simulated as well. The equilibrium model is drawn schematically in fig. 5. Equitation set 15 was used.

$$\frac{\partial P}{\partial t} = -P*(d_1 + k_1 + k_2) + T_1 * k_3$$
$$\frac{\partial T_1}{\partial t} = -T_1*(k_3 + k_4) + P*k_1$$
$$\frac{\partial T_2}{\partial t} = -T_2*d_2 + P*k_2 + T_1*k_4 \qquad (15)$$

$$Startvalues: P(0) = Bolus, T_1(0) = T_2(0) = 0$$

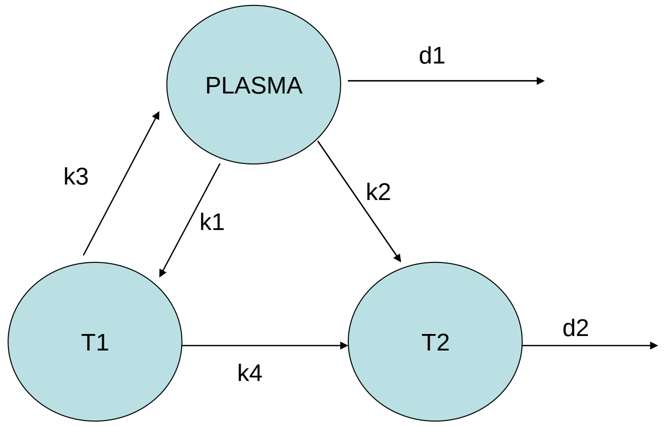

Fig. 5. Schema of equilibrium model. In this model, two tumor compartments exist. Tumor compartment 1 (T1) is in rapid equilibrium with the plasma compartment, and tumor compartment 2 (T2) accounts for slow but irreversible accumulation of PLD. The respective constants are used in equ. set 15.

This model was fitted to the data provided by [7] as well, resulting in $k_2 << k_4$ and $k_3/10.5 = k_4$.

In the equilibrium model, increased dosage and plasmapheresis after 24 h lead to an increase in peak tumor concentration of 14%. Tumor concentration rapidly drops by ~19% after plasmapheresis and AUC decreased by ~12%.

The equilibrium model, even though in contrast to the proposed features of EPR-effect, was calculated as sort of "worst case scenario" for CARL, assuming that plasmapheresis leads to a loss of already accumulated liposomes. Whether an increase in peak plasma concentration might be more important for killing tumor cells than long term AUC, can only be speculated, even though the correlation between peak plasma concentration and efficacy suggests maximum concentration as more important than AUC [6]. Even in this worst case scenario, the risk for the patient (reduction in AUC by 12%) was considered low in comparison to potential benefit by the authors and the ethics committee.

CARL-trial

TS1 was based upon a treatment schema that used PLD 35q3wks. Considering the equilibrium model, an increase in PLD dosing from 35 to 40 mg/m² was chosen for the CARL trial. Plasma-$t_{1/2}$ of PLD in mice was estimated to ~40 h [7]. With regard to plasma-$t_{1/2}$ in humans being about double that time, plasmapheresis was scheduled to ~48h after infusion of PLD. Due to certain restrictions in clinical practice and convenience for the patients, the average time between termination of infusion and plasmapheresis onset during the trial was slightly lower (46 h).
